# Discriminating Clinical and Biological Features in Malaria and Dengue Patients

**Published:** 2018-06-13

**Authors:** Wajihullah Khan, Haytham A. Zakai, Khadija Khan, Sharba Kausar, Sana Aqeel

**Affiliations:** 1 Section of Parasitology, Department of Zoology, Aligarh Muslim University, Aligarh, India; 2 Faculty of Applied Medical Sciences, King Abdulaziz University, Jeddah, Kingdom of Saudi Arabia

**Keywords:** Malaria, Dengue, Clinical features, Liver enzymes, Kidney markers

## Abstract

**Background:**

Malaria and dengue are the most widespread infectious diseases of tropical countries with an estimated 219 and 50 million cases globally. The aim of the proposed study was to find out discriminating clinical features of *falciparum* malaria and dengue.

**Method:**

*Falciparum* malaria was diagnosed by looking at the ring and gametocyte stages by microscopic examination in Giemsa stained slides. Dengue was diagnosed by ELISA for dengue-specific IgM and IgG. Liver enzymes (AST and ALT) and kidney markers (creatinine and urea) were estimated by standard biochemical techniques.

**Result:**

AST and ALT showed similar rise in both, severe malaria and dengue patients but it was much pronounced in dengue haemorrhagic fever where it attained 3–4 folds increase. Creatinine and urea showed higher levels in dengue compared to malaria. Thrombocytopenia (76.27%), convulsions (18.64%) and hepatic dysfunction (5.08%) were more prominent in dengue than that in malaria where these parameters were 50.89, 7.14 and 2.67%, respectively. Conversely, cases with anaemia, splenomegaly and jaundice were three times more in *falciparum* malaria. Acute renal failures and neurological sequelae were noticed in slightly higher number of dengue patients.

**Conclusion:**

Thrombocytopenia and hepatic dysfunction were more common in dengue, while anaemia, splenomegaly, jaundice and convulsions were more frequent in *falciparum* malaria. Neurological sequelae and cases of acute renal failure were almost equal in both the infections.

## Introduction

Dengue and malaria are the most common mosquito-borne diseases which emerged as a global public health problem. Single stranded RNA Flavivirus (DENV 1–4) is the causative agent of dengue and is generally transmitted by *Aedes aegypti* to around 50 million people of 2.5 billion population at risk throughout the world (1). Disease caused by dengue virus manifests as relatively minor febrile illness called dengue fever (DF) to a life-threatening dengue haemorrhagic fever (DHF)/ Dengue shock syndrome (DSS) (2). Malaria is another vector-borne disease caused by different species of *Plasmodium* and is transmitted by anopheline vectors in tropical countries to an estimated number of 219 million people resulting in 660,000 deaths every year throughout the world (3). Patients suffering from these diseases show somewhat similar clinical and biological presentation with variable pathological conditions (4). In malaria as well as dengue, patients suffer from high fever, headache, vomiting and severe body pain.

Majority of the dengue patients infected with flavivirus are asymptomatic but in a small proportion of cases the disease develops into the life-threatening DHF/ DSS, resulting in blood plasma leakage, bleeding and low levels of blood platelets with mortality rate of around 5% (5). Elevated values of liver enzymes were also observed in more than half of the patients in Brazil (6). Like dengue, hepatic dysfunction and renal failures were observed in patients who suffered from *Plasmodium falciparum* (7–9).

Since many symptoms and clinical features are common in *falciparum* malaria and dengue, the proposed study entitled “Discriminating clinical and biological features in malaria and dengue patients” was carried out to ascertain the response of biological markers in vital organs which could be of help in differential diagnosis of dengue and malaria.

## Materials and Methods

The present study was carried out during 2014–15 from Makkah region of Saudi Arabia and Northern zone of India based on the confirmed dengue and malaria cases. A total of 380 and 183 suspected cases were examined for the positivity of malaria and dengue, respectively.

We have used the leftover blood samples taken for diagnosis by the doctors after taking consent of the patients and ethical approval of the respective institutes.

Clinical profiles of the febrile patients were recorded at the time of their admission in the hospital. Thick and thin blood smears were prepared on glass slides by pricking the finger of the patients complaining for high fever, vomiting and headache. Thin blood films were fixed in methanol, stained with Giemsa and examined under Nikon Eclipse E-600 research microscope at x1000. Typical ring and gametocyte stages were observed for the specific diagnosis of *P. falciparum*.

Patients with fever, headache, convulsions, body ache and haemorrhagic manifestations were tested for dengue fever (DF) and dengue haemorrhagic fever (DHF). Clinical symptoms such as high fever, headache, thrombocytopenia, splenomegaly, convulsions and neurological sequelae were observed and compared in the confirmed cases of *falciparum* malaria, dengue and dengue haemorrhagic fever. For non-structural protein 1 (NS1) antigen detection, ELISA was performed in patients who had fever for 3–5d, while those who had high fever for more than 5 d after the onset of illness were tested for dengue specific IgM and IgG. Serological studies were performed with the qualitative dengue IgM capture ELISA and dengue indirect IgG ELISA (Pan Bio Ltd, Brisbane, Australia). Dengue patients were treated with acetaminophen and symptomatic therapy in the form of fluid and antipyretics whereas patients suffering from *falciparum* malaria were advised artesunate, as chloroquine and fansidar are not responding in a fairly good number of cases in our experimental areas.

Thrombocyte counts were recorded in both malaria and dengue patients. For the estimation of liver and kidney markers, blood was collected in sterile glass test tubes by vein puncture of the malaria and dengue patients after taking their consent. These samples were allowed to clot at room temperature and then transferred to refrigerator at 5 °C for 12h to squeeze the clot. Serum was collected and centrifuged at 2000rpm for 5min to remove probable contaminations and finally stored in sterilized vials at −80 °C until analyzed for liver enzymes and kidney markers. Aspartate transaminase (AST) and alanine transaminase (ALT) were estimated (10), while creatinine and urea were estimated as already reported (11, 12), respectively.

Follow up of all the patients was done till they were discharged from the hospital. All the dengue and malaria cases were monitored for their clinical, biochemical and haematological profiles. Clinical parameters of dengue and malaria positive patients were studied and compared to observe prominent discriminating features of these infections.

### Statistical analysis

All clinical parameters were examined by chi-square test using GraphPad InStat 3.06 software (GraphPad Software, Inc., California, USA), and the level of significance was set at P< 0.05. The results were expressed as mean ± standard deviation (SD) using SPSS (version 17.0, SPSS inc., Chicago, IL, USA). Graphs were plotted on Sigma plot 12.0.

## Results

### Patient characteristics

During peak season, 380 suspected cases with high fever and headache were examined for malaria from Aligarh district during 2014–15, out of which 232 (61%) were positive for malaria. Out of 232 positive cases 112 (48.27%) patients were of *P. falciparum*, while 120 (51.72%) of *P. vivax*. As for dengue is concerned, 183 suspected cases having high fever, headache and body ache were examined during its active transmission season in 2014–15 from the northeastern region of India and Makkah region of Saudi Arabia, out of which 59 (32.77%) were found positive for dengue. Majority of the patients 47 (80%) were of classical dengue, while 12 (20%) patients showed the symptoms of dengue haemorrhagic fever. Overall, 40 (67.79%) patients were found positive for IgM and 12 (20.33%) were positive for IgG. A total of 26 (44.06%) patients showed both IgM and IgG. During illness, gum bleeding and skin petechiae were seen in 3.39 and 6.80% patients, respectively.

### Symptoms in the patients

**Fig. 1.**
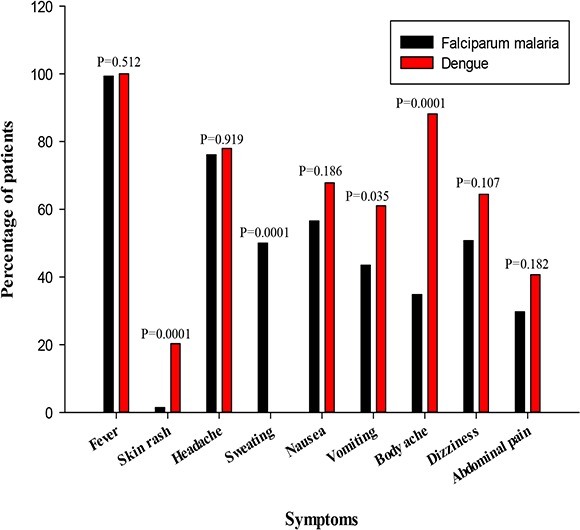
Symptoms in the patients of *falciparum* malaria and dengue

All the malaria and dengue patients had fever which ranged from 101 to 105 °C. Headache, nausea, vomiting, dizziness and joint pain were the other prominent symptoms in both *falciparum* malaria and dengue patients (Fig. 1). In this study prescribed dose of artemisinin was given to treat thirty two *falciparum* malaria patients. Parasitaemia was cleared in all the cases except 1 (3.1%), in which parasites reappeared after 23 days, indicating resistance as recrudescence occurred. Symptoms in patients infected with *P. falciparum* and dengue showed significant differences in skin rash, sweating, vomiting and body ache, but the values relating fever, headache, nausea, dizziness and abdominal pain were very close in both the diseases and did not show significant differences (Fig. 1).

### Haematological and systemic disorders

**Fig. 2.**
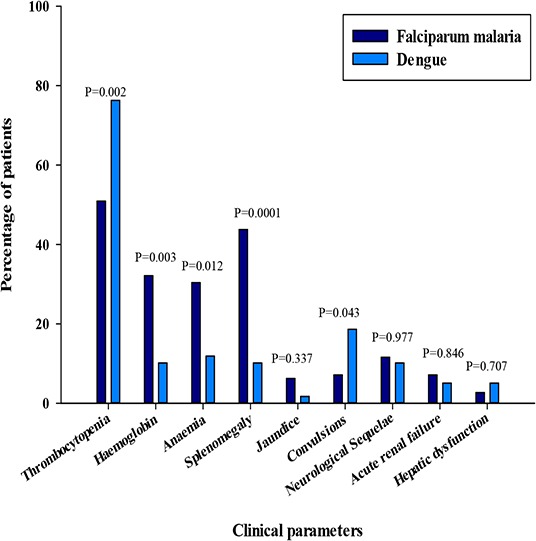
Haematological and systemic disorders in *falciparum* malaria and dengue patients

There was contrasting difference as for splenomegaly is concerned, it was 43.75% in *P. falciparum* infection compared to 10.17% of dengue showing P-value 0.0001. Thrombocytopenia in dengue patients was much higher compared to *falciparum* malaria (76.27: 50.89, P= 0.002). Convulsions and hepatic dysfunction were more common in dengue (18.64 and 5.08%) than in malaria (7.17 and 2.67%). In contrast, jaundice was more common in malaria patients (6.25%), than in dengue (1.70%). Acute renal failures and neurological sequelae were also slightly more pronounced in *falciparum* malaria compared to dengue in terms of percentage but the difference was not significant (Fig. 2). Anaemia was rarely observed in healthy individuals or patients having low or mild infections in both the diseases.

### Liver and Kidney markers

Liver damage was observed as a common complication in both malaria as well as dengue infections. Liver function test (LFT) was performed by estimating serum aspartate transaminase (AST) and alanine transaminase (ALT). The normal values of these enzymes in applied technique were 0–45IU/L and 3–60IU/L for AST and ALT, respectively. Both liver enzymes and kidney markers were almost within normal range in *falciparum* malaria and dengue fever (DF) having low to moderate infections. Slightly raised values of AST (64.70± 4.38, 98.83±22.15IU/L) and ALT (79.23±5.70, 93.83±21.15IU/L) were recorded in severe cases of *falciparum* malaria and dengue fever, respectively. Almost five times increase in the level of AST (215.50±41.71IU/L) and three times in ALT (176.00±19.79IU/L) were recorded in severe dengue haemorrhagic fever followed by mild cases of DHF where AST and ALT were recorded as 108.00±32.35 and 75.33±12.66IU/L, respectively. However, these enzymes remained within normal range in light infections.

**Fig. 3.**
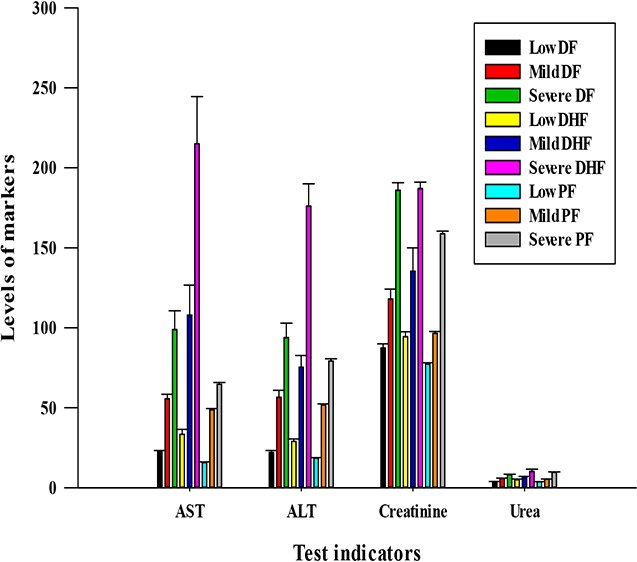
Liver and kidney markers in *falciparum* malaria, dengue and dengue haemorrhagic fever

Impairments of renal function in *P. falciparum* and dengue infections were assessed by measuring elevated creatinine and urea in serum. Patients suffering from *falciparum* malaria with high parasitemia, severe dengue and dengue haemorrhagic fever showed an increase in the level of creatinine to 158.76±6.99, 186±11.50 and 187.00±5.65μmoles/L, respectively compared to the normal range of 72–126 μmoles/L, while it was within range in low and mild infections with the exception of DHF, which showed a slightly raised value (135.33±25.38μmoles/L) in mild DHF patients. Serum urea too showed slightly higher values in severe cases of malaria, DF and DHF, which were recorded as 9.60±1.32, 8.10±0.91 and 10.20±1.97mmoles/L against the normal range of 3.0–6.0mmoles/L (Fig. 3). It was slightly raised (6.90±0.36mmoles/L) in mild cases of DHF and was within normal range in *falciparum* malaria and dengue fever.

## Discussion

Malaria and dengue are true haematological infectious diseases as they affect most of the blood components of patients. Anaemia, leucocytosis and thrombocytopenia are the commonly associated haematological complications in *falciparum* malaria, and have received much attention due to their associated mortality (13–15). In dengue and dengue haemorrhagic fever abnormalities manifest as bone marrow suppression, leucopenia and thrombocytopenia (16–18).

All the malaria, dengue and dengue haemorrhagic fever patients had high fever as earlier observed for these infections in Jazan and Makkah region of Saudi Arabia, Pakistan and India (18–21). In our study headache, nausea and vomiting were slightly more pronounced in dengue (77.96, 67.79 and 61.01%) compared to *falciparum* malaria (76.08, 56.52 and 43.47%). Headache and nausea in dengue recorded in 75 and 69% patients in Makkah region (22) were almost the same as recorded in our study. We observed vomiting in 61.01% dengue patients which was slightly less (43.47 %) in malaria patients. Earlier workers noticed vomiting in slightly less number (56%) of patients of dengue in Pakistan. Compared to malaria (34.78%), dengue patients were more frequently associated (88.13%) with body ache in the present study. In contrast body ache was recorded in only 34.44% dengue patients in Pakistan (18) which was less than half of what we observed in India and Saudi Arabia. Abdominal pain and dizziness were slightly higher in dengue patients (64.40 and 42.85%) as compared to *falciparum* malaria (50.72 and 29.71 %) in our study.

In our study, thrombocytopenia was recorded in 76.27% cases which have similarity with the earlier findings of Pakistan and India where thrombocytopenia was recorded in 60 to 79.49% dengue patients (16, 17, 20). Thrombocytopenia was less frequently associated with *falciparum* malaria and was noted in 50.89% patients in our study which is almost same as earlier observed in Pakistan where 54.5% cases of *P. falciparum* had thrombocytopenia (23). In contrast its higher value (77%) was recorded in Madhya Paradesh, India (24). Moderate degree of thrombocytopenia was observed in 26 to 50% DF cases (25). Nevertheless, deep and rapid decrease in platelet count is one of the main criteria for severe dengue (1). Thrombocytopenia was observed in DF and DHF in our study where thrombocyte counts varied from 20,000/mm^3^ to 140,000/mm^3^. Forty four percent of dengue patients had low thrombocyte counts of around 50,000/mm^3^. Continuous drop in thrombocyte count was observed by Withana et al. (26) who reported a low count of 36,000/mm^3^ on 7^th^ day of illness. It may be due to the suppression of bone marrow at an early stage which resulted in immune-mediated platelet destruction, vasculopathy and coagulopathy as earlier hypothesized (27). In fact there is an increased utilization of platelets during coagulopathy which play a major role in the induction of thrombocytopenia. Unlike dengue, thrombocytopenia was noticed in comparatively lesser number of *falciparum* malaria patients ranging between 80,000/mm^3^ and 145,000/mm^3^. It might be due to the invasion of large number of *P. falciparum* parasites which suppress bone marrow and spleen which might have resulted in immune-mediated platelet destruction which generally reflects in chronic cases.

Anaemia is an inevitable consequence in malaria due to hemolysis. We observed anaemia in 30.35% *P. falciparum* patients. In contrast, higher numbers of anaemia cases (60%) in *P. falciparum* infection were recorded in Saudi Arabia (28). Low counts for anaemia of around 13% for *P. falciparum* was also recorded in Mumbai (29). This variation might be due to severity of infection and level of immunity against the parasite in patients of *falciparum* malaria in different countries having endemic and non-endemic pockets. *Plasmodium falciparum* within the RBC modifies the erythrocytes in several ways and the cell membranes become less deformable, rigid and fragile, resulting in hemolysis and anaemia. Alteration in membranes of uninfected red blood cells by addition of glycosylphosphatidylinositol causes increased clearance of cells especially in *P. falciparum* infections that contributes to anaemia (30). Anaemia was reported only in 11.86% cases of dengue. Low rate of anaemia in dengue was probably because of a few systemic hemorrhages due to endothelial rupture as dengue virus targets monocytes or macrophages which are responsible for dissemination of virus (31).

Splenomegaly was more pronounced in *P. falciparum* infections (43.75%) compared to dengue where it was observed only in 10.17 % cases. Higher rate of splenomegaly in *falciparum* malaria was probably due to phagocytosis of parasitized R.B.Cs which got accumulated in the spleen for clearance. Almost similar enlargement in around 39–45% *P. falciparum* malaria patients was observed in Saudi Arabia (19, 32) However, much higher rate of splenomegaly (71%) was recorded in *falciparum* malaria from Rajasthan, India (33). This variation might be due to the difference in the immune status of the patients in different malaria transmission regions.

In our study, 6.25% *P. falciparum* patients had jaundice which is much less than that recorded in Sudan and Pakistan where it was reported in 14 and 43% patients (34, 35). We noticed jaundice in only 1.70% dengue patients from our study area which is much less than that in *falciparum* malaria. Slightly higher rate of jaundice (3.1%) was reported in Vietnam (36). It appears that jaundice is an outcome of the combined effects of hemolysis and liver dysfunction and probably this is the reason why jaundice was more abundant in severe cases in *P. falciparum* infections where destruction of RBC is quite high. Liver dysfunctions have been documented in earlier findings for both *P. falciparum* and dengue infections (26, 37, 38).

In our study, mean AST and ALT values were 64.70±4.38 and 79.23±5.70 in severe *P. falciaprum* cases, but the level of enzymes were within the normal range in those who had mild and low parasitaemia. Comparatively lower levels of AST and ALT (33.76–37.6 and 34.8–39.74) were recorded for mild *P. falciparum* infections in Nigeria (9, 39). In contrast, higher levels of AST and ALT (98.83±28.79 and 93.83±22.15IU/L) were recorded in the patients of dengue fever, which was still high in dengue haemorrhagic fever and was observed as 215±41.71 and 176±19.79 in our study area. Similar increase in AST and ALT was observed in dengue patients of Brazil (26). Significant rise was recorded in the level of liver enzymes in the severely infected patients as compared to mild and low infections. Liver damage in dengue infection ranges from mild lesion to fulminant hepatitis (6). It appears that the parasites during their hepatic phase destroy membrane of parasitized cells leading to leakage of the liver enzyme into the blood circulation. However, damage to hepatocytes and enzyme leakage due to toxicity of antimalarial administered to treat malaria cannot be ruled out. Increased liver enzymes may result in liver dysfunction in patients having high parasitaemia in malaria. Like malaria in dengue infections, inflammatory process in the liver parenchyma is provoked by the virus, reducing the lumen of biliary canaliculi, causing obstruction which may lead to bilirubinemia or even jaundice in a few cases (40). In dengue haemorrhagic fever hepatic injury leading to coagulopathy might have caused hemorrhage (41).

Acute renal failure (ARF) is a common cause of morbidity and mortality in severe *P. falciparum* and dengue infections. We estimated kidney markers such as creatinine and urea in both malaria and dengue patients and recorded an elevated levels of these markers in both the diseases in our experimental areas. In dengue and malaria, levels of creatinine were raised to 187±5.65 and 158.76±6.99 μmoles/L, against normal range of 72–126 μmoles/L. In severe *P. falciparum*, DF and DHF patients, urea was raised to 9.6±1.32, 8.10±0.91 and 10.20±1.97mmoles/L, respectively. We observed acute renal failures (ARF) in 7.14% *P. falciparum*. Comparatively, much higher rate of ARF of 19% was recorded from the patients in Pakistan (42). Low rate of ARF (5.08%) was observed in dengue patients of our study area in India and Saudi Arabia while earlier workers reported ARF ranging from 3.3–10.8% in Taiwan, Pakistan and India (17, 43, 44). Usually ARF occurs in non-immune individuals especially in *P. falciparum* infections with high parasitaemia, but we noticed renal failures in dengue infections as well which warrants immediate attention and change in treatment strategy especially in resistant *P. falciparum* infections. Physiological changes associated with *P. falciparum* infection make erythrocytes adherent to each other as well as to the walls of capillary vessels which cause mechanical obstructions. In addition to this, *Plasmodium* encroach and cause inflammation of glomerular capillaries resulting in altered renal microcirculation by adherent RBCs and increased accumulation of creatinine which damage the kidney tissue. This might be the probable cause of renal failures as indicated earlier (7).

Neurological sequelae are associated with damaged neurons which manifest as impaired consciousness, convulsions which often lead to coma. Cases of convulsions were more frequent in dengue (18.64%) than in malaria (7.14%) indicating more neurological damage in dengue than in *falciparum* malaria. Immune mediated mechanism and direct tropic effect of virus are implicated in neurological manifestations, as dengue antigen has been demonstrated in the brain of few patients with dengue encephalitis (45). We observed neurological sequelae in 11.60% *P. falciparum* patients. Other workers observed convulsions and other neurological deficits in 12–21% *falciparum* malaria patients of Sudan, Thailand and India (35, 46–48). Still higher rate (28%) of neurological sequelae was reported in *P. falciparum* infections in Mali (49). Like dengue, in *P. falciparum* infection too, parasites invade brain tissue and cause tropic effect on neurons which probably results in neurological deficits. We observed neurological sequelae in 10.17% dengue patients which is almost same as that of *P. falciparum*

## Conclusion

Thrombocytopenia and hepatic dysfunction were more common in dengue, whereas anaemia, splenomegaly, jaundice and convulsions were more frequent in *falciparum* malaria. Neurological sequelae and cases of acute renal failure were almost equal in both the types of infections.
